# Notes and News

**Published:** 1895-06-15

**Authors:** 


### NOTES AND NEWS.

(COLLECTED AND COMMUNICATED.)

HOSPITAL MEETINGS AND FESTIVALS, &c.

City of London Truss Society.—Festival Dinner on Friday, June 14th, at the Albion Tavern, at half-past six p.m.

North London Hospital for Consumption.—Festival Dinner on Wednesday, June 19th, at the Whitehall Rooms, Hôtel Métropole.

Paddington Green Children's Hospital.—Opening of the new hospital on Monday, July 1st, at four p.m., by H.R.H. the Duchess of Teck.

Home for Little Boys, Farningham.—Festival Dinner, Thursday, July 11th, at the Whitehall Rooms, Hôtel Métropole.

London Homœopathic Hospital.—Opening of the new buildings by H.R.H. the Duchess of Teck, on July 9th, at four p.m. Festival Dinner on Wednesday, July 10th, at the Whitehall Rooms, Hôtel Métropole.

Westminster Hospital.—Festival Dinner on Saturday, July 6th, at the Hôtel Métropole.

ALTHOUGH Whitsuntide holidays caused a lull in the Birmingham Hospital Saturday Fund collection, £12,376 has already been received.

ALTHOUGH the plague has certainly appeared at Hong Kong, the source has been easily traced, and no serious epidemic is anticipated.

M. PASTEUR has declined the decoration offered him by the German Government in recognition of his labours in the direction of science in the interests of humanity.

THE new floating hospital for infectious diseases has just been opened on the Tees. It is moored on the north side of the river, about half a mile below Eston jetty. As we have already described, when the hospital was in course of construction, it is erected on floats consisting of ten cylinders. Rolled steel girders, each 140½ feet long, connect the cylinders. The buildings consist of two ward blocks and a large administrative block midway between them, and behind the centre block are the laundry, mortuary, and destructor. The total cost of the hospital has been £100,000. The matron (Miss McCormack) showed the members of the Port Sanitary Board over the hospital, when the greatest satisfaction was expressed at the excellent manner in which the work was carried out.

THE case of a very ungrateful patient is reported in the papers. A Russian seaman, who had been attended at the fever hospital at Denton, near Gravesend, on arriving at convalescence, removed all the portable property he could lay hands on. He was kept in the hospital for a day or two out of kindness, awaiting money to be sent for his support on leaving. He decamped with the stolen goods, but was traced and apprehended.

THE Queen, in answer to an application from the Council of Charing Cross Hospital, made through the Duke of Saxe-Coburg and Gotha, K.G., president of the hospital, has presented to the Victoria Ward two prints of herself—one in her robes, and one taken at the age of 15, with the Duchess of Kent. The Victoria was the first hospital ward named after Her Majesty, and the necessary permission was granted by the Duchess of Kent in 1833.

THE plans of Messrs. Giles, Gough, and Trollope for a new asylum at Hendon have received the first prize offered by the Board of Managers of the Central London Sick Asylum District. The plans include accommodation for 250 patients—124 males and 126 females. There are to be eight general wards of 28 beds in each, besides eight small wards, one adjoining each general ward, for two beds each; and wards for lying-in cases, for noisy patients, and for measles. The site is a large one, situated at Hendon and not far from the Edgware Road.

THE first annual meeting of the Leeds Ladies' Hospital Fund has been held. It has been shown that the efforts of the ladies have been most successful, and at the same time the apprehension that the workpeople's hospital fund would suffer a diminution by the activity of the ladies' fund has not been realised. The sum collected by the ladies during the year has amounted to £757. It is proposed to distribute it between the General Infirmary, the Dispensary, the Women's and Children's Hospital, and the District Nurses' Association. We heartily congratulate Leeds on the most valuable aid which this fund offers to charities, and trust that the movement initiated here will have a rapid following throughout the country.

A FESTIVAL dinner in aid of the Cabdrivers' Benevolent Association, of which H.R.H. the Prince of Wales is patron, is to be held at the Hotel Metropole on Saturday, June 15th. The objects of the association should commend themselves to the humane public. Principal among these objects is the provision for deserving and aged and infirm cabdrivers who are broken down in health, or unable to earn their living. Amongst the men themselves, upwards of 1,000 contribute a yearly subscription of five shillings each, but as there are between 14,000 and 15,000 cabdrivers in London very considerable aid from outside is necessary to meet the requirements of the case. The association provides for sixty annuities of £20 each, and there are many other objects besides for which help is needed. Cabdrivers are especially open to temptations through the carelessness of the hirers, and it is to their credit that during a period of five years they deposited property worth £100,000 at Scotland Yard to be restored to the owners.

